# Challenges and Approaches to Recruitment for and Retention in a Dyad-Focused eHealth Intervention During COVID-19: Randomized Controlled Trial

**DOI:** 10.2196/51877

**Published:** 2024-12-03

**Authors:** Chunxuan Ma, Rachel H Adler, Daria B Neidre, Ronald C Chen, Laurel L Northouse, Christine Rini, Xianming Tan, Lixin Song

**Affiliations:** 1 School of Nursing University of Texas Health Science Center San Antonio, TX United States; 2 University of Kansas Medical Center Kansas City, KS United States; 3 School of Nursing University of Michigan Ann Arbor, MI United States; 4 Department of Medical Social Sciences Robert H Lurie Comprehensive Cancer Center of Northwestern University Northwestern University Feinberg School of Medicine Chicago, IL United States; 5 Gillings School of Global Public Health University of North Carolina at Chapel Hill Chapel Hill, NC United States; 6 School of Nursing Mays Cancer Center University of Texas Health Science Center at San Antonio San Antonio, TX United States

**Keywords:** randomized controlled trials, RCT, prostate cancer, accrual, retention, COVID-19 pandemic, family-based research

## Abstract

**Background:**

Family-based randomized controlled trials (RCTs) encounter recruitment and retention challenges. Cancer-focused RCTs typically recruit convenience samples from local cancer centers and hospitals.

**Objective:**

This study aimed to examine the recruitment and retention of a population-based, patient-partner dyad cohort in an RCT testing a dyadic eHealth intervention to improve the quality of life in patients with prostate cancer and their partners.

**Methods:**

In this 2-arm, parallel-group RCT, men who recently completed treatment for localized prostate cancer statewide were recruited through North Carolina Central Cancer Registry rapid case ascertainment between April 2018 and April 2021, coinciding with the COVID-19 pandemic. Patient-partner dyads underwent baseline assessments and were randomly assigned to either the intervention or control groups. Follow-up surveys were conducted at 4, 8, and 12 months after baseline. Descriptive and logistic regression analyses were used to achieve the study’s aims.

**Results:**

Of the 3078 patients referred from rapid case ascertainment, 2899 were screened. A total of 357 partners were approached after obtaining the eligible patients’ permission, 280 dyads completed baseline assessments and were randomized (dyad enrollment rate: 85.11%, 95% CI 81.3%-88.9%), and 221 dyads completed the 12-month follow-up (retention rate: 78.93%, 95% CI 74.2%-83.7%). Regarding the factors associated with retention, compared with White participants, people self-reporting as “other races” (including American Indian, Asian, and multiracial) were more likely to drop out of the study (odds ratio 2.78, 95% CI 1.10-7.04), and older participants were less likely to withdraw (odds ratio 0.96, 95% CI 0.92-0.99).

**Conclusions:**

Despite the negative impact of the pandemic, we successfully recruited enough patient-partner dyads to test our RCT hypotheses. Our recruitment and retention rates were equivalent to or higher than those in most dyadic intervention studies. A well-functioning research team and specific strategies (eg, eHealth intervention, internet phone, and online surveys) facilitated the recruitment and retention of patients with prostate cancer and their partners during the unprecedented pandemic.

**Trial Registration:**

ClinicalTrials.gov NCT03489057; https://clinicaltrials.gov/study/NCT03489057

**International Registered Report Identifier (IRRID):**

RR2-https://doi.org/10.1186/s13063-021-05948-5

## Introduction

Participant recruitment and retention for cancer survivorship research have always been innately challenging for family-focused research, especially for randomized controlled trials (RCTs), due to the geographical distance between participants and the trial location [1]; patients’ poor functional status, high symptom burden, and health deterioration [2,3]; and caregiver unavailability [4]. Most studies have used convenience samples of patients with cancer and caregivers recruited from one or a few cancer centers, hospitals, and community settings, leading to small sample sizes and low generalizability of study results [4].

Social isolation during the COVID-19 pandemic compounded the difficulties with family-based intervention RCTs, especially the challenges of participant recruitment and retention [5-7]. Long-term “shelter in place” orders negated in-person strategies and forced researchers and participants to use online or telehealth options to continue research studies [8,9]. More importantly, for older adults and patients who are immunocompromised, like many patients with cancer and chronic illnesses, isolation was imperative to protect their health. Supportive care for patients with cancer also changed with the pandemic, from in-person meetings and interactions to becoming partially or solely reliant on online resources [10,11]. These issues have brought critical challenges to the recruitment and retention of adult patients with cancer and their partners in family-based RCTs.

We conducted a dyadic RCT (ClinicalTrials.gov NCT03489057, principal investigator LS) that innovatively used North Carolina Central Cancer Registry (NCCCR) rapid case ascertainment (RCA) to recruit a population-based group of patients and their partners from across the state. In this RCT, we test the efficacy of a couple-focused eHealth intervention, titled the Prostate Cancer Education and Resources for Couples (PERC) program, which provides online education and skills training, an online forum for professional and peer support, and local and national resources for symptom management after cancer treatment for patients with prostate cancer and their intimate partners. However, the RCT timeline coincided with the COVID-19 pandemic, which introduced significant barriers to cancer diagnosis, treatment, and care [12]. Patients with prostate cancer are usually older adults (median age 66 years) with multiple comorbid conditions. They are at an increased risk for COVID-19 infection and prone to the adverse effects of the COVID-19 pandemic–related health problems. These characteristics complicated the already challenging recruitment and retention of patients with prostate cancer and their partners. This study examines the challenges, strategies, and outcomes of the RCT recruitment and retention of patients with prostate cancer and their intimate partners during the pandemic.

## Methods

### Study Design

We used a 2-arm, randomized, repeated measures, longitudinal design to test the efficacy of the eHealth PERC intervention among survivors with localized prostate cancer and their intimate partners [13]. We randomly assigned the patient-partner dyads who completed baseline (T1) surveys to either the intervention group (PERC) or the control group (National Cancer Institute [NCI] prostate cancer website). Dyads then completed follow-up surveys at 4 (T2), 8 (T3), and 12 (T4) months after baseline. All research activities were conducted virtually, through telephone and email, and the PERC intervention itself was a web-based program (see our protocol for details) to reduce the burden for patients, their partners, and the research team in this family-based RCT [13]. After the onset of the pandemic, we started to use a HIPAA (Health Insurance Portability and Accountability Act)–compliant, cloud-based phone system to contact study participants remotely. We also changed in-person weekly team meetings to Zoom (Zoom Video Communications) virtual meetings to continue our discussion of program-related issues and develop appropriate strategies to enhance recruitment, retention, and intervention fidelity.

### Participants

Patients were eligible if (1) they were English-speaking men aged 40-75 years who were within 15 weeks of completing treatment with curative intent for their localized prostate cancer (ie, surgery, radiation therapy with or without hormonal therapy); (2) they did not have another cancer diagnosis within the previous 2 years; and (3) they were not receiving current treatment for another form of cancer. We only included the eligible patients if they had an intimate partner willing to participate in the study. Intimate partners were recruited if they were English speakers over 18 years old, had no previous diagnosis of cancer themselves, or had not received cancer treatment within the last year. Patients and their partners were excluded from the study if either exhibited severe cognitive impairment.

### Recruitment Process

We recruited patient-partner dyads through the NCCCR RCA [14]. RCA accelerates the regular reporting timeline for the NCCCR; it allows researchers to identify incident patients with cancer within 1 week of diagnosis. This RCT targeted the 36 counties with the highest proportions of ethnic populations and people living in poverty to maximize the study’s ability to recruit a diverse cohort. RCA staff screened the electronic medical records at cancer centers and hospitals in these counties and identified patients who were 40-75 years old and had been diagnosed with nonmetastatic prostate cancer.

Institutional review board (IRB) approval was obtained from the University of North Carolina-Chapel Hill before any research activities. Following the principal investigator’s (LS) relocation, we obtained IRB approval from the University of Texas Health Center at San Antonio for the analysis of deidentified data from the RCT. The NCCCR sent consent information to the potentially eligible patients and informed them about the PERC RCT. RCA staff then extracted the cancer-related data (diagnosis, time of diagnosis, stage, and biopsy results) and contact information of patients and their health care providers. RCA staff uploaded these data to an encrypted, password-protected data transfer hub for the research team to download biweekly; the trained research assistants downloaded and transferred the data to REDCap (Research Electronic Data Capture; Vanderbilt University), a password-protected, secure, encrypted, HIPAA-compliant database for data entry and management [15]. Within a total of 6 weeks after RCA staff sent the patients’ NCCCR information, the research assistants mailed study information packages (including a descriptive introductory letter, brochure, and informed consent information) to each patient and his physician, giving each group up to 3 weeks to opt out of the RCT before they screened the prospective patients and partners. The project coordinator monitored recruitment, and the research assistants tracked recruitment and other research activities using REDCap.

### Enrollment Process

Using the university-designated phones, team email, and their own emails, the research assistants assessed the eligibility of patients and partners using screening surveys and answered any questions; patients and partners who met the inclusion criteria provided consent separately. The informed consent was completed through telephone; audio recorded; and saved in an encrypted, password-protected database separate from the REDCap surveys. The research assistants then scheduled the patients and partners to complete their baseline (T1) survey separately. Automated reminder emails and voice mails were sent to consented dyads on days 3 and 5 if they still needed to answer T1 survey phone calls. On day 14, a research assistant sent a personal follow-up email. After patients and partners completed the informed consent and T1 survey, the dyads were randomly assigned to the PERC or NCI control group (1:1). These administrative and survey data were entered into the REDCap separately. The survey data were deidentified using an assigned unique ID number linking to the identifiable tracking administrative database, which only authorized study personnel could access.

Administering the surveys by telephone was initially the only planned remote data collection option. We added the online survey options after the onset of the pandemic in response to requests of the participants for more flexibility in completing the surveys due to increased demands at work and home. The biostatistician assistant of the study helped research assistants generate personalized URLs using the REDCap online survey panel; research assistants emailed the URL to the enrolled participants. Enrolled patients and partners could choose to complete either a phone or online survey throughout the remainder of the study.

### Retention Process

Patients and their partners separately completed the follow-up surveys after dyads completed the PERC or use of the NCI prostate cancer website at 4, 8, and 12 months after baseline (T2, T3, and T4, respectively). Two weeks before a follow-up survey was due, the study staff sent a reminder letter to dyads’ homes with a survey answer key example; the study staff then called the participants up to 12 times during a 4-week period, rotating morning, afternoon, and evening during weekday and weekends, to schedule survey completion through the phone or online. If patients or partners preferred to complete the online survey, the study staff sent the link in 3 automated emails on days 0, 3, and 6 (at T2, T3, and T4). We considered the dyads who did not respond to any previous contact attempts by day 60 as “lost to follow up” and discontinued contact. To ensure privacy and confidentiality, all phone calls were behind closed doors in private rooms. Research assistants and coordinators used group emails and their personal secured university emails to contact participants.

We offered each patient and partner gift cards of US $20, US $30, US $30, and US $50 for completing the surveys at T1, T2, T3, and T4, respectively, with up to US $130 in financial incentives for completing the entire study. In addition, we sent each participant retention gifts, that is, a fanny and a duffel bag at 6 and 10 months, respectively. We also sent postcards to each dyad over the holidays (eg, Thanksgiving, Christmas, and New Year’s Day).

Retaining patient-partner dyads during the 4-month PERC intervention (or use of the NCI website) was just as crucial as follow-up survey completion. The interventionist was a master’s degree–prepared nurse clinician with decades of experience working with patients with genitourinary diseases, including prostate cancer. In preparation for her work on the study, she received 20 hours of PERC intervention training. REDCap automatically notified the interventionist through email after a dyad was randomized to the PERC or NCI control group. Patients and partners received their usernames and passwords for a central study website through email and mail, which blinded the study participants and the research staff. After logging in, the dyads were directed to either the NCI prostate cancer web page or the PERC website. The research staff provided ongoing technical assistance.

In addition, we informed participants in both groups that they could contact the study team at any time (including the interventionist) through telephone (including a toll-free number), email, and the “Contact Us” link on the study website. The research team responded to participants’ inquiries and requests within 24-48 hours. The team also sent all participants study updates, including midterm outcome findings or publications, through email (the details about the PERC intervention and the NCI control can be found in our protocol paper [13] and outcome paper that is forthcoming).

### Measurements

#### Outcomes

To calculate the enrollment and retention rates, we obtained the numbers of participants who refused or withdrew from the study, as well as those who enrolled and remained in the RCT, after exporting and deidentifying the relevant administrative data in REDCap.

#### Enrollment Rate

We defined the enrollment rate as the number of dyads enrolled (both the patient and partner consented and completed) at T1 divided by the number of dyads contacted and accepted (neither the patient nor partner declined). In this dyadic study, dyads were not considered enrolled if one partner consented and completed T1 but the other partner did not consent, or if 1 partner consented without completing T1 by the time the other partner consented and completed T1.

#### Retention Rate

We defined the retention rate at each follow-up as the number of dyads who completed corresponding surveys divided by those who enrolled.

#### Other Variables

We collected Gleason scores from RCA referral documents and categorized the patients into low-to-intermediate (Gleason score<8) and high grades (Gleason score8) [16]. Race and comorbidity were collected separately from patients’ and partners’ self-reports at baseline. We categorized the race as White, Black, and others (including American Indian, Asian, and multiracial). Comorbidity was measured using the Charlson Comorbidity Index (CCI). CCI calculated one’s comorbidity score by summing up the comorbid conditions the individuals reported as “yes” [17].

### Data Analyses

We conducted descriptive analyses to examine the recruitment and retention rates and presented the 95% CI values for these rates. Chi-square tests assessed differences in survey completion (phone vs online) among patients and their partners, as well as overall response rates at follow-ups T2 to T4. Logistic regression was used to explore the impact of demographic and clinical factors—specifically, age, race, CCI of patients and partners, and patients’ Gleason scores—on retention, while adjusting for group assignment. The Mann-Whitney *U* test was used to examine age differences between participants opting for online versus telephone surveys at each assessment point (T1, T2, T3, and T4). All tests were conducted at a 2-sided significance level of α=.05, using SAS software (version 9.4, SAS Institute Inc.), and all confidence intervals are 95% CIs.

### Ethical Considerations

The study was approved by the Institutional Review Boards at the University of North Carolina at Chapel Hill (IRB# 17-0482) and the Institutional Review Boards at the University of Texas Health Science Center at San Antonio (IRB# 23-0191). The team obtained written or verbal consent from all patients with prostate cancer and their partners enrolled in this study. The verbal consent was recorded. Written and verbal consent was saved in a password-protected secured folder on an encrypted network separate from the survey data. Only authorized research staff had access to the recordings and data.

## Results

### Overview

The mean age of the patients was approximately 64 years, and their partners’ age averaged 61 years. Their average relationship length was nearly 33 years. While all patients were male, most partners (279/280, 99.6%) were female. Approximately 76.79% (215/280) of patients identified as White patients, and 23.21% (65/280) identified to Black or other racial groups. About 70% (196/280) of patients had less than a college or bachelor’s degree, with nearly 49% (135/280) reporting a family income exceeding US $90,000. More than half of the patients and partners (127/280, 45.36%) were not presently working. Approximately three-quarters of patients (208/280, 74.29%) underwent surgery.

### Recruitment

Between April 2018 and April 2021, the NCCCR RCA referred 3078 patients with prostate cancer who were 40-75 years old and potentially had a partner, as indicated in their electronic medical record. The RCA staff uploaded an average of 49 referrals every other week between April 2018 and December 2019 in the prepandemic period and 31 referrals from January 2020 to April 2021 during the COVID-19, representing a 36.73% decrease due to the reduced number of patients receiving active treatment because of the closure and lockdown of health care facilities.

**Figure 1:**
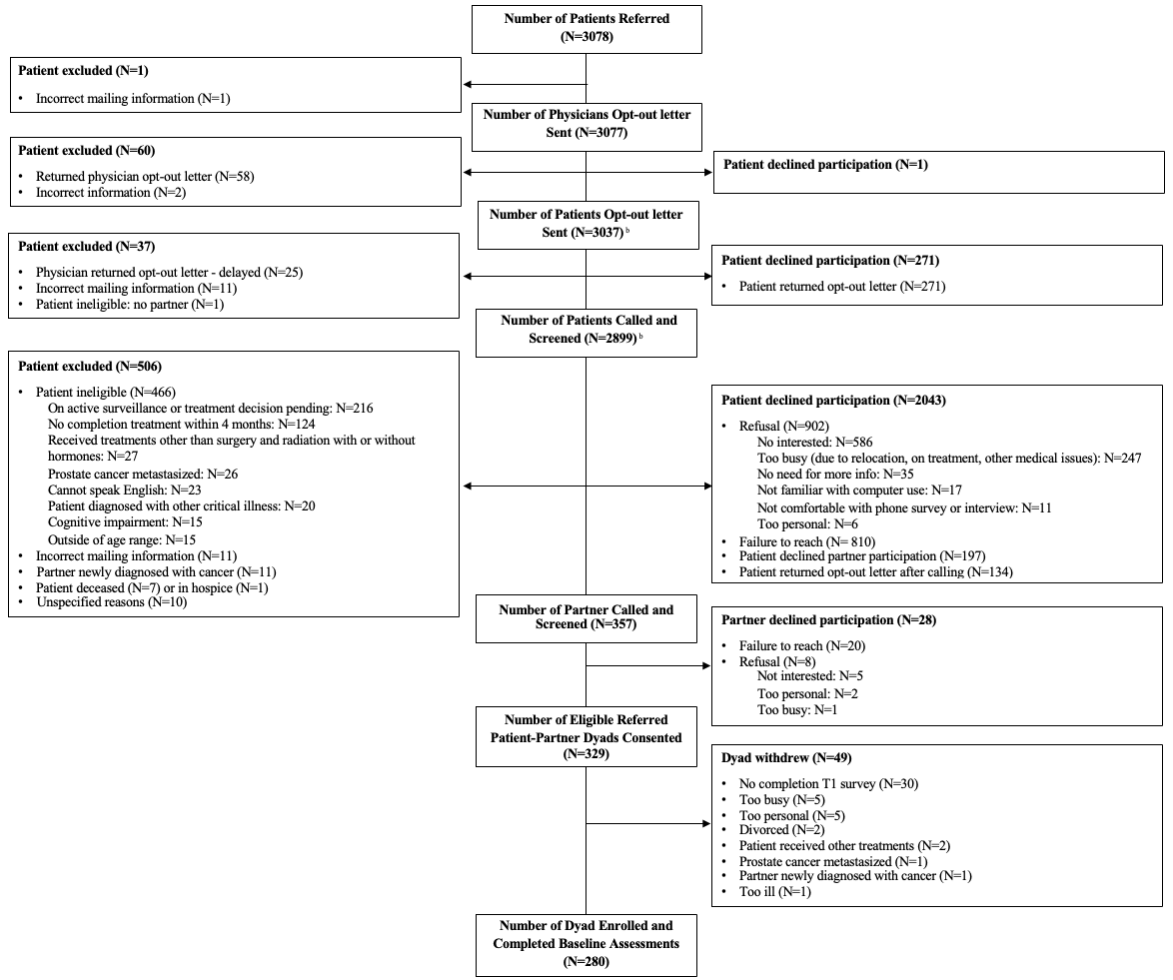
CONSORT patient recruit flow diagram. Our study focuses on both patients and partners. *Discrepancies in the dyads' completion number at each time point were due to dyads contacting our team to skip 1 or 2 follow-up surveys. CONSORT: Consolidated Standards for Reporting Trials.

The research team sent 3077 physician opt-out letters (1 patient was removed due to incorrect mailing information) and screened 2899 prospective patients for eligibility (Figure 1 [18]). We successfully contacted the partners of 357 prospective patients who met the inclusion criteria and provided us with their partner’s contact information. In total, we excluded 2721 patients from the RCA patient referral pool for reasons such as refusal (ie, opt-out letters returned: n=83, 3% physicians and n=405, 14.9% patients; invalid contact information: n=835, 30.7%; and ineligibility: n=507, 18.6%).

Among the 357 patient-partner dyads contacted, we obtained consent from 329 dyads after 28 partners declined participation because of failure to reach (n=20), lack of interest (n=5), too personal (n=2), and having no time (n=1). A total of 49 dyads withdrew after consenting because they did not complete the baseline survey (n=30), were too busy (n=5), felt the study was too personal (n=5), or were divorced (n=2). The other drop-out reasons also included the patient having received new treatment (n=2) or cancer metastasized (n=1), the partner having a new cancer diagnosis (n=1), or the patient being too ill (n=1; Figure 1).

### Enrollment

**Figure 2:**
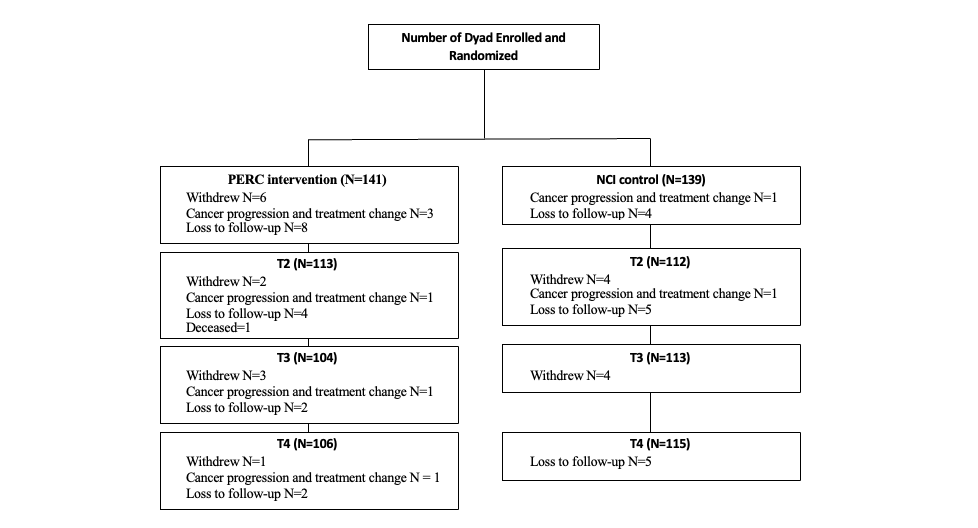
CONSORT dyads recruit and follow-up flow diagram. CONSORT: Consolidated Standards for Reporting Trials; NCI: National Cancer Institute; PERC: Prostate Cancer Education and Resources for Couples.

**Figure 3:**
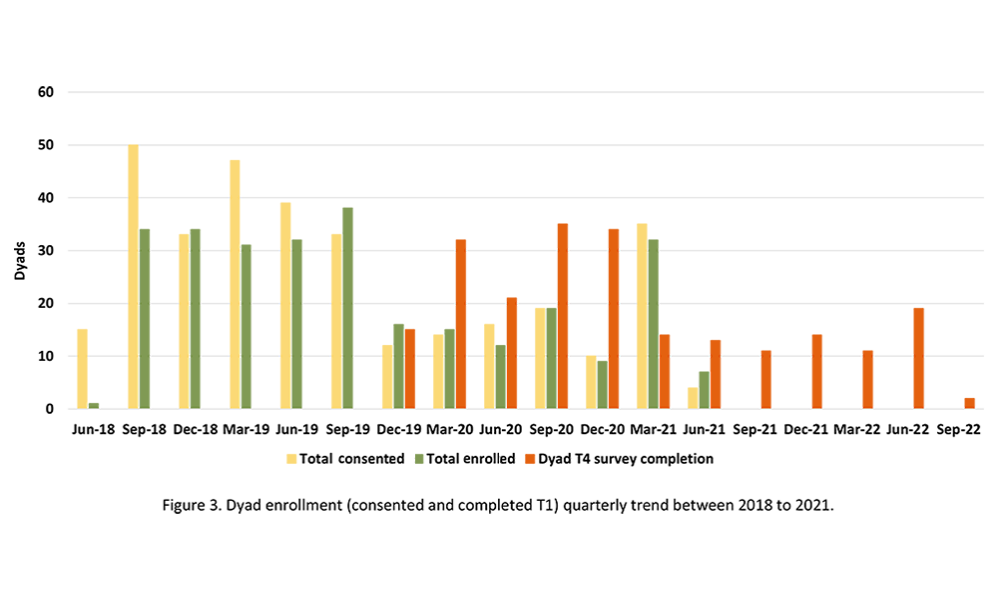
Dyad enrollment (consented and completed T1) quarterly trend between 2018 and 2021.

As displayed in Table 1 and Figure 2, we enrolled 280 patient-partner dyads, including 1 same-sex couple, 20.7% (58/280) Black dyads, and 2.5% (7/280) Asian and Native American dyads. The mean age of enrolled dyads was 63.08 (SD 7.25) years. We randomly assigned the dyads to the PERC intervention (n=141) or the NCI control group (n=139). The demographics were reported in our previous paper [19]. Among the 3078 referred patients, 11.6% (n=357) were eligible and consented to the research team to contact their partners*.* Our dyad enrollment rate was 85.1%, that is, the number of dyads who consented and completed T1 (n=280) / (number of dyads contacted [n=357] – number of dyads refused [n=28]). The trend in the number of consented and enrolled dyads is presented in Figure 3.

**Table 1:** Recruitment and retention by race and group.

Group		Total, N	White^a^, n (%)	Retention rate, %	Black^a^, n (%)	Retention rate, %	*P* value^b^	Others, n (%)	Retention rate, %
**RCA** ^c^ **referred**		3078	1992 (64.72)	—^d^	696 (22.61)	—	—	390 (12.67)	—
**Enrolled**		280	215 (76.79)	—	58 (20.71)	—	—	7 (2.5)	—
**T1**									
	PERC^e^	141	107 (75.89)	—	28 (19.86)	—	—	6 (4.26)	—
	NCI^f^ attention control	139	108 (77.7)	—	30 (21.58)	—	—	1 (0.72)	—
**T2**									
	PERC	113	88 (77.88)	82.24	24 (21.24)	85.71	.78	1 (0.88)	16.67
	NCI attention control	112	85 (75.89)	78.7	26 (23.21)	86.76	.44	1 (0.89)	100
**T3**									
	PERC	104	83 (79.81)	77.57	19 (18.27)	67.86	.29	2 (1.92)	33.33
	NCI attention control	113	86 (76.11)	79.63	26 (23.01)	86.76	.44	1 (0.88)	100
**T4**									
	PERC	106	82 (77.36)	76.64	22 (20.75)	78.57	.83	2 (1.98)	33.33
	NCI attention control	115	88 (76.52)	81.48	26 (22.61)	86.76	.60	1 (0.87)	100

^a^We used the patient’s race to represent the dyad because there was high congruence in patient and partner self-reported races: 92.3% (205/215) of partners of White patients identified as White, and 91.4% (53/58) of partners of Black patients identified as Black.

^b^*P* value obtained from Fisher tests and chi-square tests. The difference between White and other races was not calculated because of the small sample size. A *P* value of .05 is considered significant.

^c^RCA: rapid case ascertainment.

^d^Not available.

^e^PERC: Prostate Cancer Education and Resources for Couples.

^f^NCI: National Cancer Institute.

### Retention

Figure 2 presents a CONSORT (Consolidated Standards for Reporting Trials) diagram of randomization and follow-up surveys [18]. Our retention rates were 80.36%, 77.5%, and 78.93% at T2, T3, and T4, respectively. The T2 and T3 retention rates were lower than expected (~90% and 85% at T2 and T3, respectively), whereas the T4 retention rate was close to expected (80% at 12 months after baseline). There was no racial difference in participant retention rates at different time points (Table 1).

**Figure 4:**
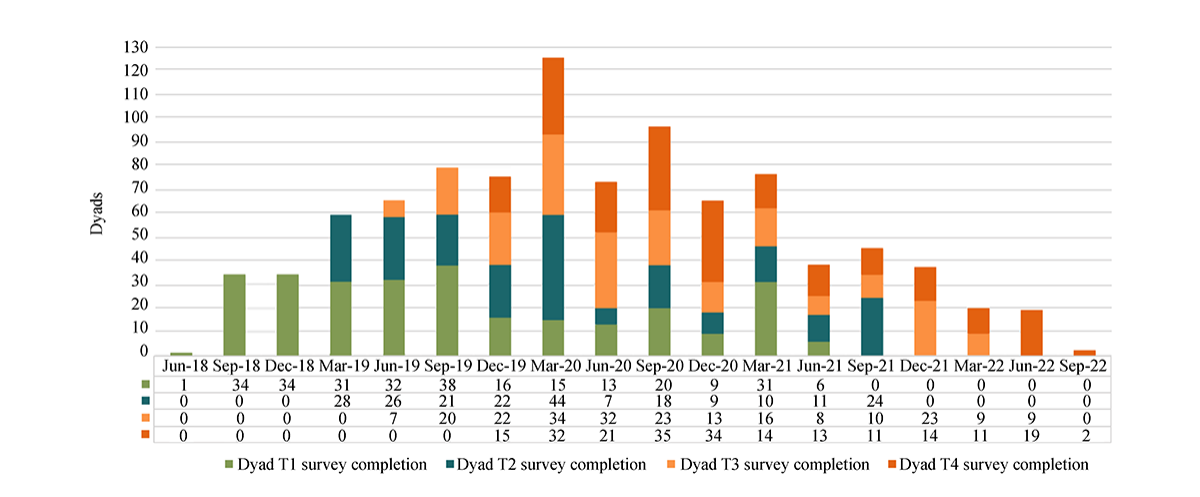
Dyads T1 to T4 completion (every 3-calendar months during 2018-2022).

Furthermore, 94/280 dyads (33.6%), 128/225 dyads (56.9%), 168/217 dyads (77.4%), and 185/200 dyads (92.5%) completed the baseline T1, T2, T3, and T4 surveys, respectively, during the COVID-19 pandemic (January 2020 to March 2022). The total number of completions peaked in the first 3 months of 2020 and subsequently fluctuated, but with an overall downward trend (Figure 4). The percentages of participants who chose the online versus telephone surveys increased significantly throughout the study (*P*<.001): 23.93% (134/559), 34.89% (157/450), 57.14% (248/434), and 73.3% (324/441) for T1, T2, T3, and T4 (Table 2), respectively. There is a significant age difference between participants who used phone versus online surveys at T1, T2, and T3; participants of older age were more likely to complete phone surveys at T1 to T3 (*P* values <.01), but no age difference was found at T4. Compared with White participants, people self-reporting as “other races” (including American Indian, Asian, and multiracial) were more likely to drop out of the study (odds ratio 2.78, 95% CI 1.10-7.04), and older participants were less likely to withdraw (odds ratio 0.96, 95% CI 0.92-0.99).

**Table 2:** Frequency of survey type by group.

Group and role		T1^a^ (n=559), n (%)					T2 (n=450), n (%)					T3 (n=434), n (%)				T4^a^ (n=441), n (%)		
		Phone survey		Online survey		Phone survey			Online survey		Phone survey		Online survey		Phone survey			Online survey
**PERC** ^b^																		
	Patient	105 (18.75)	36 (6.43)		71 (15.78)			42 (9.33)		38 (8.76)			66 (15.21)	27 (6.11)			78 (17.65)	
	Partner	104 (18.57)	37 (6.61)		70 (15.56)			43 (9.56)		38 (8.76)			66 (15.21)	25 (5.66)			81 (18.33)	
**NCI** ^c^																		
	Patient	108 (19.29)	30 (5.36)		78 (17.33)			34 (7.56)		54 (12.44)			59 (13.59)	32 (7.24)			83 (18.78)	
	Partner	108 (19.29)	31 (5.54)		74 (16.44)			38 (8.44)		56 (12.9)			57 (13.13)	33 (7.47)			82 (18.55)	
**Total**		425 (75.89)		134 (23.93)		293 (65.11)			157 (34.89)		186 (42.86)		248 (57.14)		117 (26.47)			324 (73.3)

^a^Participants completed surveys using mixed methods (half phone survey and half online survey) at T1 (n=1) and at T4 (n=1).

^b^PERC: Prostate Cancer Education and Resources for Couples.

^c^NCI: National Cancer Institute.

## Discussion

### Principal Findings

To our knowledge, the PERC study is the first to use a cancer registry RCA to facilitate the recruitment of a population-based sample of patient-partner dyads who have been recently treated for prostate cancer for a symptom management eHealth intervention RCT. We completed participant recruitment and follow-up data collection despite the COVID-19 pandemic. We received 3087 referrals from RCA. Following a thorough screening process, we obtained permission from 357 eligible patients to contact their partners for consent. Subsequently, 280 patients and partners consented, completed baseline assessments, and were randomized, achieving an enrollment rate of 85.11%. Among these, 221 dyads completed the 12-month follow-up, reflecting a robust retention rate of 78.93%. Previous reviews have indicated that 86% of RCTs fail to finish on time, mainly due to insufficient enrollment [20]. Patient participation in RCTs decreases with advanced age, and ethnic and minority groups are underrepresented [21]. Coupled with the difficulties inherent to family research, as discussed previously, these challenges indicate that patients with prostate cancer, with a median age of 66 years, are among the hardest-to-reach populations. The outbreak of the COVID-19 pandemic in 2020 and the subsequent shelter-in-place for extended periods further complicated the dyad-focused PERC RCT. Despite these challenges, we proactively and adaptively used a series of strategies, including contacting participants using telephone, email, and regular mail; using telephone and online surveys; and implementing an eHealth intervention program, to ensure completion of the proposed study as planned. Although significantly impacted by the COVID-19 pandemic lockdowns, we successfully accrued enough study participants (280 dyads) to test our RCT hypotheses; our enrollment and retention rates in this NCCCR RCA–facilitated, dyad-focused, eHealth RCT are superior to the family-based intervention studies that recruited from cancer centers, hospitals, and community settings [4]. We also found that people who were younger and who self-reporting as other races were more likely to drop off from the RCT as compared with their White counterparts, and older participants were more likely to complete surveys through phone during T1, T2, and T3.

### Participant Recruitment

Our study is unique in that we used a cancer registry to recruit patient-partner dyads for a symptom management eHealth intervention RCT. Cancer registries have been used extensively in cancer surveillance and epidemiologic research [22,23] and in research that aims to improve cancer detection and treatment [24-26], mostly among patients with cancer [22-26] and occasionally among patients and clinicians [27]. We used a novel NCCCR RCA–facilitated approach to expand our geographic catchment area and enroll patients with newly treated prostate cancer and their intimate partners. To facilitate the recruitment of participants across the state of North Carolina and to reduce costs, we targeted the 36 counties with the highest proportions of ethnic populations and the highest rates of poverty and designed our protocol to contact patients through phone, text messaging, email, and ground mail, without in-person meetings. Upon the onset of the pandemic, we quickly adjusted the work schedule for our research team members to adapt to the lockdown orders and shelter-in-place mandates without affecting the overall flow of the study or requiring significant protocol amendments. As a result, we continued to recruit throughout the pandemic and successfully enrolled 280 prostate cancer patient-partner dyads. Our enrollment rate of prostate cancer patient-partner dyads (85.11%) is significantly higher than the mean and median enrollment rates (33% and 23%, respectively) of studies recruited from cancer hospitals and centers using in-person and clinician referral approaches [4]. The racial composition of our study participants (215/280, 76.79% White) is similar to that (75.9%) of family-based intervention studies conducted in the United States and the United Kingdom [4].

Although recruitment using the NCCCR RCA allowed us to overcome many of the challenges when in-person contact was impossible during the pandemic, we excluded approximately 43% (1331/3096) of the referred patients because of incorrect contact information and patient ineligibility, indicating a need to improve the accuracy of data in electronic medical records and during data extraction (eg, updated contact information, diagnosis, and treatment). Researchers could benefit from working closely with the cancer registrars to refine initial screening criteria and reduce the referral of ineligible patients.

We have identified the reasons for nonparticipation in this dyad-focused eHealth RCT. Like previous patient-focused RCTs [21,28], the main reasons for nonparticipation included a lack of interest, privacy concerns about discussing personal issues, and challenges related to patients’ cancer and treatment options. It is worth noting that 197 patients declined partner participation, making them ineligible for this dyad-focused RCT. Prostate cancer is known as a “couple’s illness” because cancer-related symptoms and stress negatively affect the quality of life of patients and their partners, and partners often have worse quality of life than the patients [29]. Dyad-focused interventions and RCTs have become increasingly common in managing health issues and quality of life challenges for patients with cancer or their caregivers (eg, partners); this is because the improvement in symptoms and quality of life involves psychosocial processes and communication between patients and caregivers, and resolving complex issues require couples to work together as a team [30-32]. When patients act as gatekeepers and decline partner participation, patients and partners both miss the opportunity to engage in and benefit from a dyad-focused symptom management program. Future research is needed to understand why patients decline their partners’ participation and develop strategies to engage them, as members of dyads, in psychosocial behavioral interventions to improve both people’s quality of life. Future RCTs can also stratify randomization based on partner availability.

It is worth noting that due to the intimate and private nature of prostate cancer, the privacy of cancer-related information and hesitancy to discuss topics of a personal nature were central challenges for many dyads. Regardless of assurances from the study team and explanations of measures taken to protect privacy and confidentiality, many patients and partners in the recruitment phase refused to participate over concerns about access to their personal, medical, and psychosocial information. During the RCT, we noted participant hesitancy or refusal to discuss intimate issues such as sexual function or sexual symptoms. Researchers must consider participants’ desire to maintain privacy and confidentiality, earn their trust, and engage in best practices for addressing their legitimate privacy concerns during recruitment and throughout study implementation, especially when in-person contact is impossible.

### Improve Communication and Build Trust During Recruitment and Retention

Because trust is the foundation of recruitment and retention [33], especially in digital health research [34], we used different strategies to build trust among dyads in the RCT. Although providing flexibility and convenience, contacting prospective participants through telephone, email, and mail without face-to-face meetings could jeopardize participant accrual and retention, because cold-calling and remote contacts reduced trust. The sensitive nature of prostate cancer could exacerbate these concerns for patients and partners. Furthermore, prospects and participants may not have felt the continued desire to communicate or ask questions as time passed since treatment completion and as the pandemic continued. In this RCT, we adopted the following strategies built upon previous research [35-37] to acknowledge the vital role of trust and communication in recruitment and retention (Textbox 1).

**Strategies to acknowledge in recruitment and retention.**

- We provided opt-out letters for both the patients and their physicians, which enhanced patient autonomy (eg, offering choice and control), beneficence (eg, reducing selection bias), and justice (eg, reducing lack of generalizability) [38].
- We formed a diverse research team that comprised members of different demographic backgrounds (eg, Black, Asian, and White American; Hispanics and non-Hispanics; and young and older males and females) so that patients and partners felt comfortable communicating with the research team. We included pictures of and information about the research team members on the study website hosted at a research University and in the physician and patient opt-out letters to help develop familiarity with the team members.
- We provided extensive training for the research staff and used role play to practice interviews for recruitment, consent, and surveys.
- When contacting participants, our staff used the university-designated team email and their email accounts through their dedicated university office phone numbers and, later a Health Insurance Portability and Accountability Act–compliant, cloud-based phone system, which displayed the University of North Carolina name; hence, participants knew who was calling them.
- We ensured that each participant was contacted for all surveys by the same research staff throughout the study period unless participants requested a different staff member. Doing so helped the research staff and study participants become familiar with each other, have consistent interpersonal interaction, and build rapport; this made the participants willing to share their concerns and feel comfortable requesting scheduling changes rather than canceling the research activities altogether.
- To ensure participant convenience, we asked their preferred time for being contacted and accommodated mornings, afternoons, and evenings during both weekdays and weekends.
- We responded to participants’ phone calls and emails within 24-48 hours. Research staff contacted clinicians and coinvestigators for assistance when they could not answer the questions or address participants’ concerns.
- We planned regular reminder emails, follow-up emails, and phone calls consistent with the recruitment schedules, follow-up surveys, and intervention activities.
- We provided gift cards after each survey and retention gifts with our team logo between follow-up surveys.

These approaches were vital to maintaining positive communication and establishing trust with our participants, from our early recruitment stage, when we helped prospective participants develop an interest in the study, to follow-ups when participants were not responsive. Consistently using these strategies ultimately led us to achieve our recruitment and retention goals for this dyad-focused eHealth intervention RCT despite the extended period of the stressful pandemic. Despite these strategies having been used to improve the representation of minority participants in previous research [35-37], our study population included one same-sex dyad. The overall percentages of Black American (58/280, 20.71%,) and other racial group patients (7/280, 2.5%, mainly Asian and Native American) in our RCT are also slightly lower than those in the North Carolina population (22.1% and 3.7%, respectively) [39]. The high incident and mortality rates of COVID-19 among Black and Asian participants might have negatively affected the participation of racially diverse minority participants because the percentage of Black patients dropped from >25% before COVID-19. Future research must explore more effective, rigorous strategies to promote racial diversity in family-based intervention research.

### Participant Retention

A positive influence on our retention rates was that in our protocol, the method of participant contact was flexible, as we contacted participants through phone, text messages, email, and ground mail. Although before the pandemic, many studies used in-person or phone surveys and interviews [33], we built more flexibility into our method of study implementation (eg, surveys conducted through telephone and later online). What is significant to participant retention is that the PERC intervention is an online platform. We intentionally developed PERC to address the needs of patients who live in rural areas and to accommodate the private nature of prostate cancer symptoms and complications (eg, sexual dysfunction). The online medium provides patients and family caregivers easy access to posttreatment supportive care whenever and wherever they need it. In addition, we provided training for participants unfamiliar with online platforms. We loaned participants a tablet computer and hotspot internet connectivity if they could not access the internet.

Meanwhile, the pandemic and associated lockdowns changed the research environment as the public became familiar with online technology for social interactions, meetings, and schooling during the pandemic. Thus, as a result, online surveys have become more widely used in research [40]. Many of our participants also requested more flexibility in completing survey assessments due to increased demands at home and work. After team discussion and consultation with our psychometrician, we added the online survey option to meet participants’ needs. The percentage of participants who completed online surveys increased significantly over time, ranging from 23.93% (134/559) for T1 to 73.3% (324/441) for T4. Unlike other RCTs for which follow-up retention rates decrease over time, our retention rates at different time points were flat (ie, 80.36%, 77.5%, and 78.93% at T2, T3, and T4, respectively). Adding the online survey option might have eased the negative impact of pandemic-related chaos and stress on our participants’ completion of study surveys during 2020 and early 2021, which might have reduced the effects of the pandemic lockdown on the RCT. It is worth noting that our retention rates were similar in both the PERC and control groups across different time points for different racial groups; these retention rates are equivalent to or better than most family-based intervention studies (69%-70% on average) [4].

### Limitations

This study has several limitations. First, we excluded a large percentage of the patients referred by the cancer registry RCA because of incorrect contact information and ineligibility, which may have caused sample selection bias. Second, the percentages of Black American patients and patients from other non-White groups were lower than projected despite the various strategies we used. Anecdotally, some dyads from minority groups declined RCT participation because the partners worked multiple jobs while the patients were out of work due to prostate cancer and related treatment. Future research must examine effective, innovative strategies to reduce system barriers and engage nonintimate caregivers from minority groups. Finally, verifying participants’ responses to online surveys was difficult when they entered incorrect data. We ensured data quality and accuracy for the telephone survey by randomly selecting up to 50% of all data to check against the phone survey recording and conducting double data entry. For the online surveys, our database manager immediately examined the data after the surveys were submitted; we contacted the respondents if the data were incomplete or included unusual values. Despite controversies in the strengths and weaknesses of online versus telephone surveys [41,42], online surveys have been shown, as compared with telephone surveys, to produce more reliable and complete data and to be cheaper and less time-consuming to conduct [42]. We provided free internet access (ie, iPad [Apple] and JackPot [Microgaming]) and training to increase the participation of people with limited ability to engage in digital health research. Following the recommendation for recruiting a diverse study population [43], we combined telephone and online surveys to ensure complete and correct data reporting, especially during the unprecedented disruption of the COVID-19 pandemic. Finally, the retention efforts of the study team, while effective for the RCT, may not translate well to intervention implementation when the team may not be available for participant engagement. However, many strategies can help enhance communication between the research team and participants, build trust, and ultimately, benefit participant enrollment and retention in family-based research projects.

### Conclusions

This study details the recruitment and retention challenges of our dyad-focused eHealth intervention RCT, related strategies that our team used, and outcomes of participant recruitment and retention during the pandemic. We innovatively used the NCCCR RCA to identify potential participants, which enabled us to continue to recruit during the COVID-19 pandemic, as we could complete the RCT without in-person human contact. Similarly, our use of online surveys, eHealth intervention, and other alternatives to in-person interactions, as well as our dedication to persistent and regular communication with participants, positively participant recruitment and retention during the unprecedented pandemic.

### Data Availability

Data supporting the findings of this study are available from the corresponding author upon reasonable request.

## Appendix

Multimedia Appendix 1 CONSORT-eHEALTH checklist (V 1.6.1).

## Abbreviations

CCI: Charlson Comorbidity Index
CONSORT: Consolidated Standards for Reporting Trials
HIPAA: Health Insurance Portability and Accountability Act
IRB: institutional review board
NCCCR: North Carolina Central Cancer Registry
NCI: National Cancer Institute
PERC: Prostate Cancer Education and Resources for Couples
RCA: rapid case ascertainment
REDCap: Research Electronic Data Capture
RCT: randomized controlled trial
